# Actin filaments mediated root growth inhibition by changing their distribution under UV-B and hydrogen peroxide exposure in *Arabidopsis*

**DOI:** 10.1186/s40659-020-00321-3

**Published:** 2020-11-23

**Authors:** Meiting Du, Yanhong Wang, Huize Chen, Rong Han

**Affiliations:** 1 Higher Education Key Laboratory of Plant Molecular and Environmental Stress Response, Shanxi Normal University in Shanxi Province, Linfen, 041000 Shanxi China; 2 School of Life Sciences, Linfen, 041000 Shanxi China

**Keywords:** Abiotic signaling, Actin dynamics, UV-B, Root morphogenesis

## Abstract

**Background:**

UV-B signaling in plants is mediated by UVR8, which interacts with transcriptional factors to induce root morphogenesis. However, research on the downstream molecules of UVR8 signaling in roots is still scarce. As a wide range of functional cytoskeletons, how actin filaments respond to UV-B-induced root morphogenesis has not been reported. The aim of this study was to investigate the effect of actin filaments on root morphogenesis under UV-B and hydrogen peroxide exposure in *Arabidopsis.*

**Results:**

A Lifeact-Venus fusion protein was used to stain actin filaments in *Arabidopsis*. The results showed that UV-B inhibited hypocotyl and root elongation and caused an increase in H_2_O_2_ content only in the root but not in the hypocotyl. Additionally, the actin filaments in hypocotyls diffused under UV-B exposure but were gathered in a bundle under the control conditions in either Lifeact-Venus or *uvr8* plants. Exogenous H_2_O_2_ inhibited root elongation in a dose-dependent manner. The actin filaments changed their distribution from filamentous to punctate in the root tips and mature regions at a lower concentration of H_2_O_2_ but aggregated into thick bundles with an abnormal orientation at H_2_O_2_ concentrations up to 2 mM. In the root elongation zone, the actin filament arrangement changed from lateral to longitudinal after exposure to H_2_O_2_. Actin filaments in the root tip and elongation zone were depolymerized into puncta under UV-B exposure, which showed the same tendency as the low-concentration treatments. The actin filaments were hardly filamentous in the maturation zone. The dynamics of actin filaments in the *uvr8* group under UV-B exposure were close to those of the control group.

**Conclusions:**

The results indicate that UV-B inhibited *Arabidopsis* hypocotyl elongation by reorganizing actin filaments from bundles to a loose arrangement, which was not related to H_2_O_2_. UV-B disrupted the dynamics of actin filaments by changing the H_2_O_2_ level in *Arabidopsis* roots. All these results provide an experimental basis for investigating the interaction of UV-B signaling with the cytoskeleton.

## Background

UV-B light is an inherent component of sunlight that strongly affects plant development [1, 2]. The UV-B photoreceptor UVR8 (UV resistance locus 8) is required for UV-B responses in plants [3]. In the absence of stimulation by UV-B, the UVR8 protein is mainly distributed in the cytoplasm. After receiving a certain intensity of UV-B, UVR8 accumulates in the nucleus quickly, with the entire process completed in only a few minutes [4]. The UVR8 protein directly and rapidly binds to COP1 (Constitutively Photomorphogenic 1) in the nucleus, and this depends on UV-B. After UVR8 binds to COP1, the expression of the transcription factor HY5/HYH (Elongated Hypocotyl 5/HY5 Related Homolog) is initiated. In addition, UVR8 can also interact with multiple transcription factors, such as WRKY36 (WRKY DNA-binding protein 36), BIM1 (BES1-interacting MYC-like 1), and BES1 (BRI1-EMS-suppressor 1), to directly regulate gene expression [5]. Recently, researchers found that BR signaling inhibits UV-B stress responses in *Arabidopsis thaliana* and various crops by controlling flavanol biosynthesis [6].

UV-B inhibited *Arabidopsis* root growth, and UVR8 was also expressed in the root [7]. Recently, it was confirmed that the UV-B photoreceptor UVR8 interacts with MYB73/MYB77 (MYB domain protein 73/77) to regulate auxin responses and lateral root development [7]. UVR8-dependent UV-B perception occurs mainly in the epidermis and cortex, but deeper tissues such as the endodermis can also contribute [8]. UV-B exposure triggers rapid ROS (reactive oxidative species) production in *Arabidopsis* root apical cells, which are involved in the general acceleration of endocytic vesicle recycling [9]. However, vesicle cycling mainly depends on the dynamics of actin filaments [10]. This implies that it is necessary to study the function of actin filaments in roots.

The actin cytoskeleton is a complex network of protein filaments extending through the cytoplasm of cells. Actin filaments can be highly dynamic and reorganize continuously when the cell shape changes or in response to environmental cues [11]. In mammalian cells, the nucleus and its genomic content may be organized by intranuclear actin filaments, particularly with regard to a given stage of the cell cycle [12]. Recent results showed that AN (Angustifolia) and ACTIN7 regulate centripetal nuclear positioning in *Arabidopsis* leaves. This suggested that the AN-DYRKP complex regulates the alignment of actin filaments during centripetal nuclear positioning in leaf cells [13].

However, an increasing number of studies have revealed the key functions of actin filaments throughout the cytoplasm of plant cells. The actin cytoskeleton in the plant cytoplasm is a highly dynamic structure that mediates various cellular functions, in large part through accessory proteins that control the balance between monomeric G-actin and filamentous actin since actin filaments are essential for cytoplasmic organization [14]. Actin filaments mediate the movement of chloroplasts in *Arabidopsis* [15]. Their dynamics function in vesicle transport during rapid and directional pollen tube growth in *Arabidopsis* [16]. Additionally, actin organization in a growing root hair, with fine actin filaments in the apical region and thick actin bundles further down the base of the tube [17], affects vesicular trafficking. Actin filaments participate in mitosis in the wheat root meristem region [18] and affect the the influence of growth hormone on root growth. The C-terminal headpiece domain of Villins is important for exerting the inhibitory effect of TIBA (Auxin transport inhibitor, 2,3,5 triiodobenzoic acid) on the actin cytoskeleton and auxin transport in plants [19]. Cytokinins promote actin bundling and cell elongation by activating the signaling pathway involving cytokinin receptors [20].

Compared to this well-known function of actin filaments, less is known about the effects of second messengers on the organization of actin filaments. Calcium (Ca^2+^) serves as a versatile intracellular messenger that has been shown to regulate actin dynamics and rearrangements in plants [21]. Nitric oxide (NO) is a small bioactive signaling molecule that modulates actin filament organization in *Arabidopsis* primary root cells at low temperatures [22]. ROS have emerged as major regulatory molecules in plants and play roles in early signaling events initiated by environmental stimuli [23]. The dynamics of actin filaments in *Arabidopsis* are disrupted under salt stress due to the regulation of ROS levels [24]. However, few detailed studies on the mechanism by which ROS affect the dynamics and distribution of actin filaments in roots have been reported. Although researchers have also raised questions about ROS and actin filament reassembly, little progress on this topic has been made so far [25].

Previous results showed that UV-B promotes actin filament bundle formation in wheat protoplasts and induces abnormal chromosome movement [26]. Recently, it was reported that light was channeled through the stem to the roots [27], and UV-B signals could be transmitted to the roots through plant endodermis tissues [8]. Whether the inhibition of root growth by UV-B is related to actin filaments is an interesting question that is worth studying.

Here, we found that hypocotyl elongation and root growth in *Arabidopsis* were inhibited by exposure to UV-B in either Lifeact-Venus (Col-0) or *uvr8* plants. In addition, H_2_O_2_ was detected only in the root region but not in the hypocotyl under UV-B exposure. Confocal microscopy results showed that the actin filaments were partially disaggregated, and the alignment changed in the inhibited hypocotyl under UV-B exposure. The in vitro application of H_2_O_2_ inhibited root growth, with changes in actin filament dynamics at different concentrations. The dynamics of actin filaments in the control root under UV-B exposure showed the same tendency observed after excess H_2_O_2_ treatments, which was very different from the dynamics observed in the *uvr8* root.

These results indicated that the inhibition of hypocotyl elongation under UV-B exposure changed the arrangement of actin filaments, which was not related to H_2_O_2_ production. However, the inhibition of root growth was considered to be a result of the H_2_O_2_ burst, which in turn affects actin filament depolymerization and reassembly under UV-B exposure. Overall, our results provide an experimental basis for further understanding the functions of actin filaments, which could include serving as downstream functional molecules in UV-B signaling.

## Materials and methods

### Plant growth and growth conditions

The Columbia ecotype of *Arabidopsis thaliana* was used. The 35S-promoter GFP- fABD2 (fimbrin actin-binding domain 2) fusion construct [28] and Lifeact-Venus fusion construct [29] allowed in vivo visualization of the dynamic actin filaments. *uvr8* (SALK_033468) crossed with Lifeact-Venus and T2 plants were used for visualization. For every transformation, more than 10 independent transgenic lines with a single copy of the transgene were generated. Seeds were sterilized in 10% (v/v) bleach, placed on 1/2 MS medium containing 0.8% (w/v) agar and 1% (w/v) sucrose, and stratified for 4 days at 4 °C in the dark before being transferred to white light or white light plus UV-B (Philips TL20W/01RS narrowband UV-B tubes, 2 W/m^2^, measured by a TENMARS-213 UV Light Meter) [7]. Exogenous H_2_O_2_ was added to 1/2 MS medium to prepare different H_2_O_2_ treatments before the sterilized seeds were placed on top. For the DMTU (dimethylthioureurea) treatment, 7 DAG seedlings on a 1/2 MS plate (+ H_2_O_2_) were transferred to a 1/2 MS plate (+ 5 mM DMTU and H_2_O_2_) [30], regrown until 16 DAG and captured for phenotype analysis. For analysis of root length, images of seedlings grown on vertically orientated 1/2 MS plates were captured with a camera (Cannon 70D), and measurements were made with a ruler. In each condition, 25–30 seedlings were analyzed for each experiment.

### Fluorescence staining

An aliquot of H2DCF (Thermo Fisher) was added to all treatments to obtain a final concentration of 5 μM. After 15 min, the samples were washed 3 times with PBS (pH = 7.4) to remove excess H2DCF and visualized immediately using an Olympus FluoView FV1000 confocal microscope. Three independent experiments were performed.

### Confocal fluorescence imaging analysis

Cellular fluorescence was monitored using an FV-1000 confocal system with a 1.4 NA, 60 × oil immersion objective lens. Cell observations were made for at least three replicate samples. GFP, Venus, and H2DCF were excited at 488 nm and detected at 515–530 nm. H_2_O_2_ production was quantified in the root cells by measuring the fluorescence intensity of H2DCF. Three independent experiments were conducted, and 15 roots were used in each experiment. The fluorescence intensity of H2DCF was calculated using ImageJ software. The integrated density was quantified using cortical sections of root cells. Integrated density was determined using the respective analysis option in ImageJ [31]. To measure the fluorescence intensity (mean value) of the actin cytoskeleton, a rectangle of 1000 μm^2^ was drawn in each figure, and a mean of 5–10 cells per condition were analyzed. For every treatment, a minimum of 50 cells were considered. To quantify H_2_O_2_- or UV-B-mediated changes in cytoskeletal organization, we measured the skewness of Lifeact-Venus-marked actin filaments. For that measurement, z-stacks of entire cells were acquired and subsequently processed with ImageJ. All z-stack images were skeletonized and projected, and the skewness of the actin filaments, which indicates the degree of actin bundling, was measured as described previously [32].

### Statistical analysis

Data results are expressed as the means ± standard deviations (SD). Statistical significance was assessed using one-way analysis of variance (ANOVA) with a general linear model, followed by Tukey’s test (p < 0.05), using SPSS 21.0 and Sigma-plot 10.0.

## Results

### Distribution of actin filaments in different transgenic lines

**Fig. 1:**
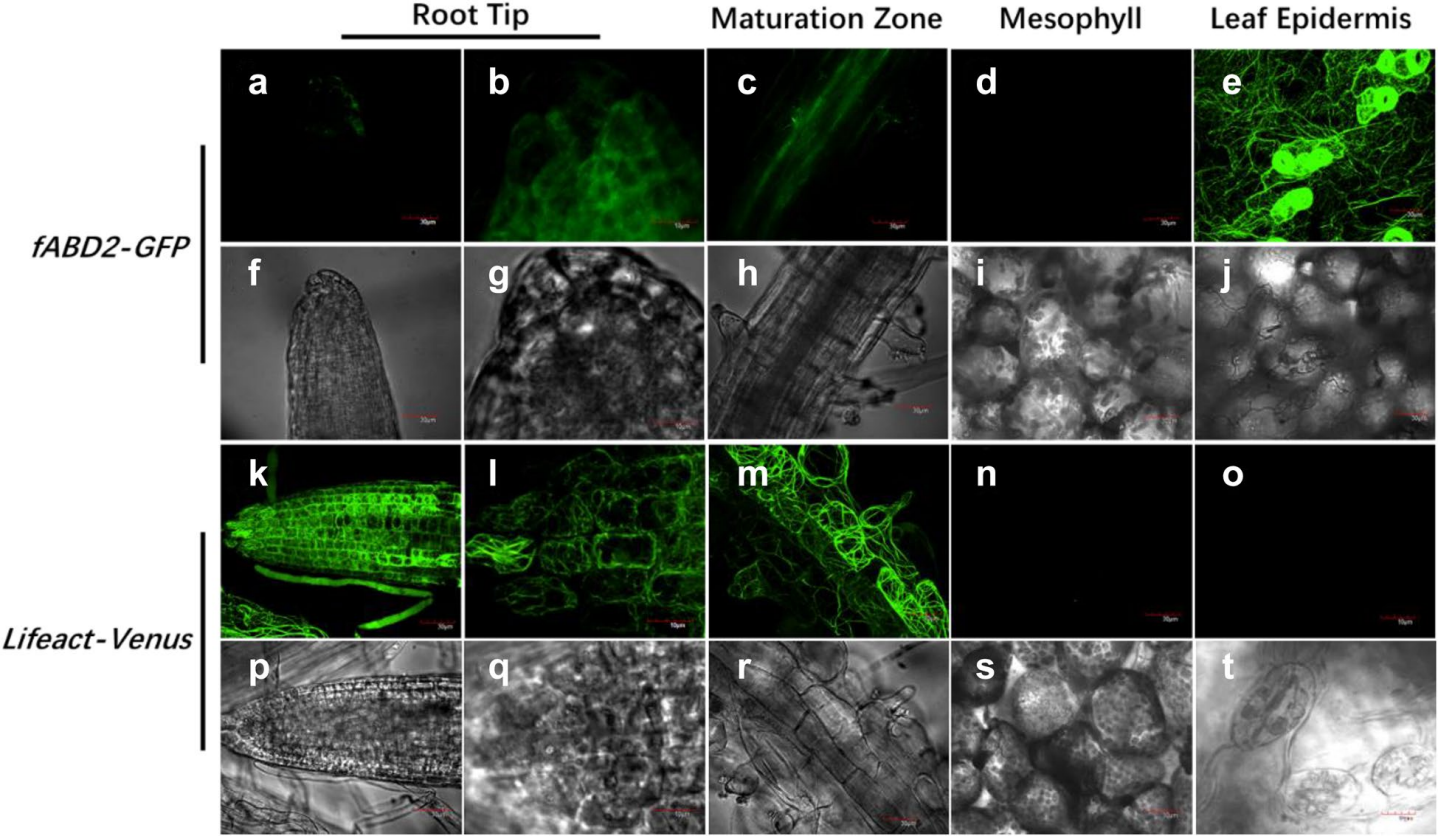
Observation of different actin filament marker lines in *Arabidopsis.* **a**–**e** The GFP signals in different regions of *fABD2-GFP* transgenic *Arabidopsis*. **f**–**j**. The DIC channel of panels **a**–**e**. **k**–**o** The Venus fluorescence in different parts of the Lifeact-Venus* Arabidopsis* line. **p**–**t** The DIC channel of panels **k**–**o**. Scale bars = 10 μm (**b**, **g**, **l**, **q**); 30 μm (all panels except **b**, **g**, **l**, **q**)

Since we focused on the response of actin filaments to environmental factors in *Arabidopsis* thaliana roots, we tried to select a transgenic line that could be used to better observe actin filaments in roots from the *fABD2-GFP* and Lifeact-Venus transgenic lines. The 7 DAG root tip, mature root regions, mesophyll cells, and leaf epidermal cells of these two lines were observed, as shown in Fig. 1. For the *fABD2-GFP* line, weak green signals appeared in different tissues of the seedling (Fig. 1a–e), but the strongest signal was in the leaf epidermis (Fig. 1e). For the Lifeact-Venus line, the green signal was much stronger in the root region than in any other parts of the seedling, and we could not detect a fluorescence signal in mesophyll or epidermal cells (Fig. 1n, o). Therefore, considering the subject matter of this study, the Lifeact-Venus transgenic line was chosen as the experimental line.

### UV-B inhibits hypocotyl elongation and root growth of Arabidopsis seedlings

**Fig. 2:**
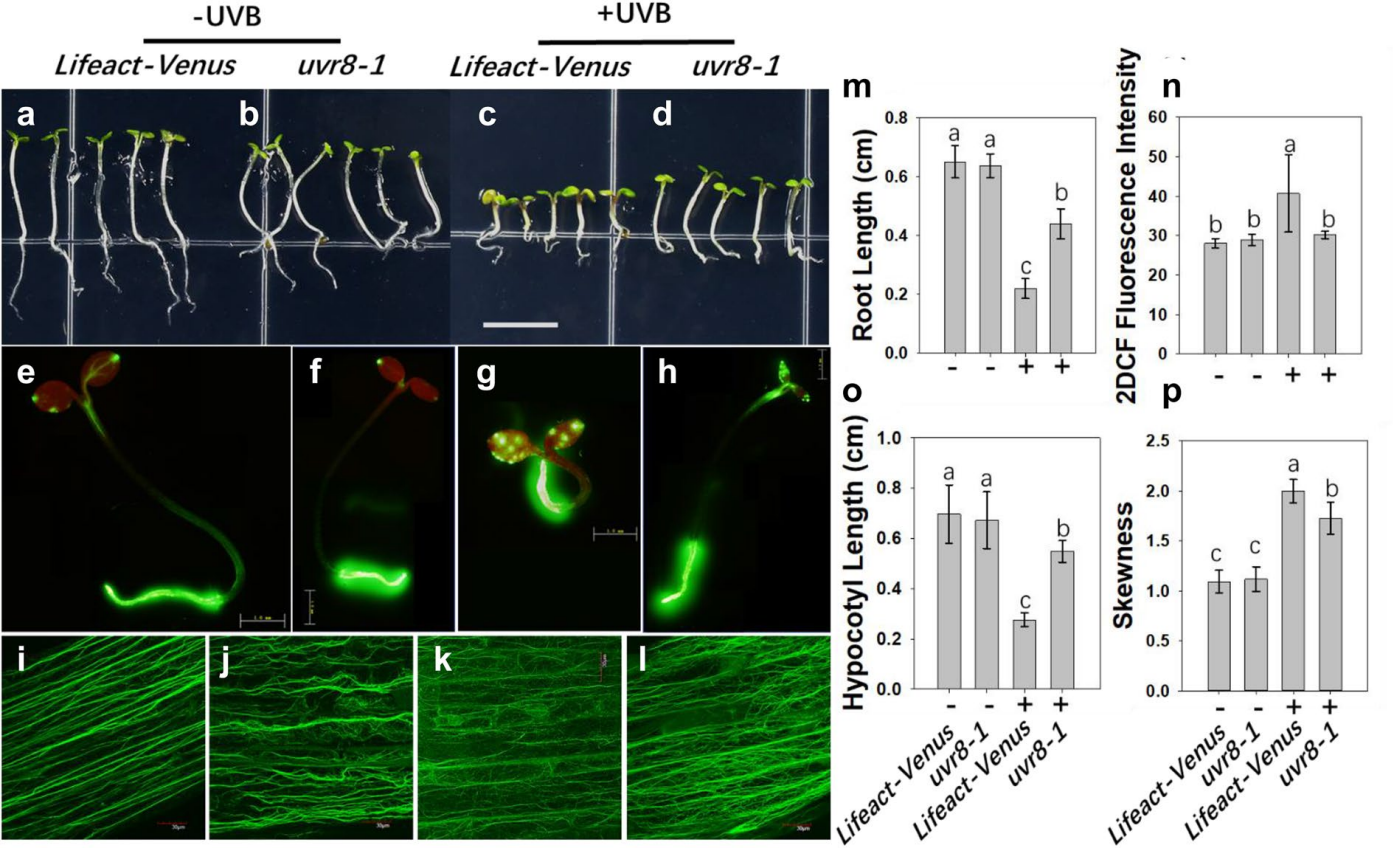
UV-B inhibited hypocotyl elongation and root growth in *Arabidopsis* seedlings. **a**–**d** UV-B inhibited the hypocotyl elongation of 5 DAG seedlings of Lifeact-Venus and *uvr8*. **e**–**h** H_2_O_2_ content in the roots after H2DCF staining. **i**–**l** The distribution of actin filaments in the *Arabidopsis* hypocotyl before and after UV-B treatment in Lifeact-Venus and *uvr8*. **m** The effects of UV-B on *Arabidopsis* root length. **n** Statistics on the mean fluorescence intensity of H2DCF staining in roots from each group. **o** The effects of UV-B treatment on hypocotyl elongation. **p** Statistics on skewness in hypocotyls of each group. Scale bar = 5 mm (**a**–**d**); 10 mm (**e**–**h**); 30 μm (**i**–**l**). Different letters indicate significant differences (*P* < 0.05).

Hypocotyl elongation was inhibited after UV-B exposure in Lifeact-Veuns and *uvr8* seedlings*,* as shown in Fig. 2a–d. The reduction in the length of the hypocotyl in the UV-B-treated group was ~ 63.3% in the control group and 20.9% in the *uvr8* group (Fig. 2o). UV-B exposure also inhibited root growth, as shown in Fig. 2m. Compared with that of the untreated plants, root length was reduced by ~ 68.2% in the control group and 33.8% in the *uvr8* group. H2DCF (Fig. 2e–h) staining showed that the H_2_O_2_ concentration in the root increased sharply after UV-B treatment in the Lifeact-Venus line but not in the *uvr8 line.* After calculating the H2DCF fluorescence intensity (Fig. 2n), it was found that UV-B treatment resulted in a 30.1% increase in H_2_O_2_ content in the Lifeact-Venus line. Confocal microscopy observations revealed that the dynamics of actin filaments were changed, and the shape converted from a bundle to a much thinner arrangement in the less elongated hypocotyl in the Lifeact-Venus line after exposure to UV-B (Fig. 2i, k). The *uvr8* line also showed reorganization of actin filaments in the shortened hypocotyl under UV-B exposure (Fig. 2j, l). The orientation of the actin filament bundles changed from parallel to the hypocotyl axis to perpendicular to the elongation of the hypocotyl axis under UV-B exposure. The skewness of actin filaments increased substantially under UV-B in both the Lifeact-Venus line and *uvr8* lines (Fig. 2p). These results indicated that the inhibition of hypocotyl elongation by UV-B exposure may be due to changes in the distribution of actin filaments. This change was independent of ROS since the ROS burst was not detected in the hypocotyl.

### Exogenous H_2_O_2_ inhibited root growth in Arabidopsis

**Fig. 3:**
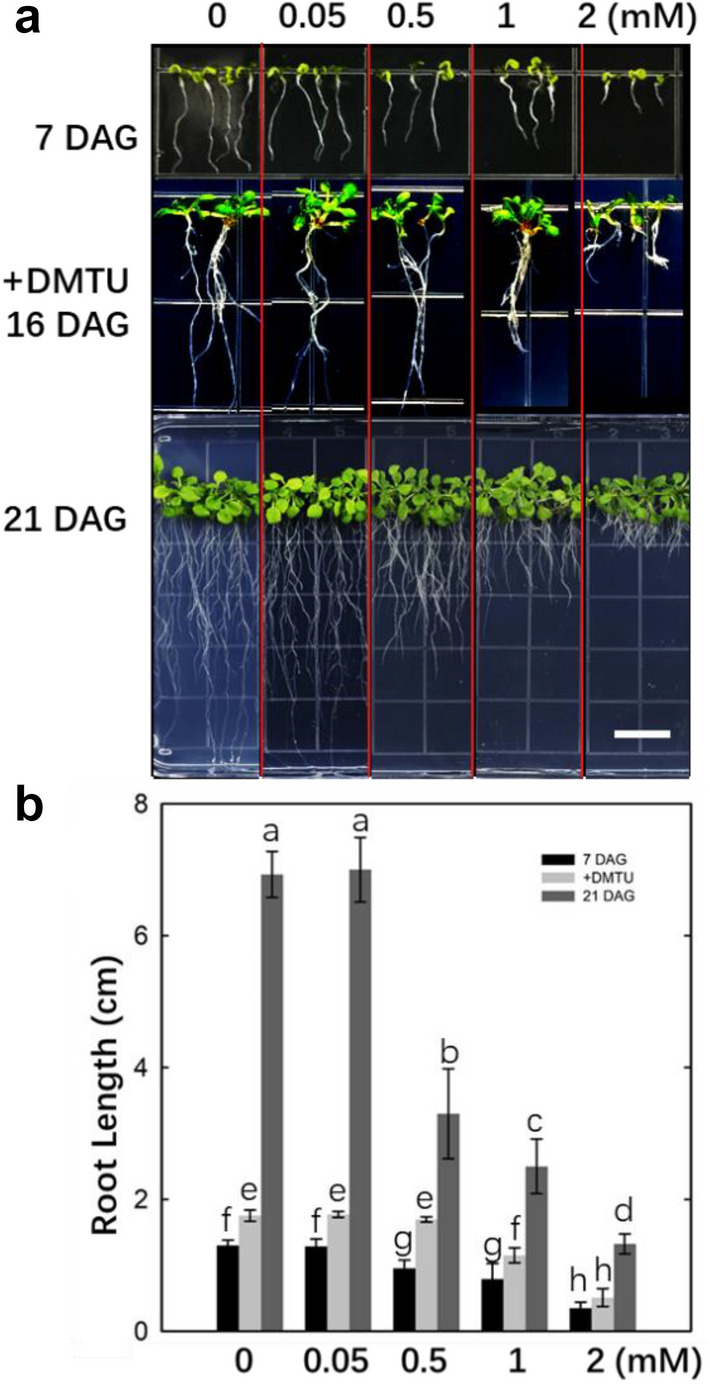
Effects of exogenous H_2_O_2_ application on* Arabidopsis* root growth. **a** The inhibitory effects of different concentrations of exogenous H_2_O_2_ on the growth of *Arabidopsis* seedlings at 7 DAG and 21 DAG and the recovery effects of DMTU on *Arabidopsis* seedlings at 16 DAG. **b** The root length results after different treatments. Scale bar = 10 mm (**a**). Different letters indicate significant differences (*P* < 0.05)

Previous results showed that UV-B caused an increase in ROS in Arabidopsis roots, especially an increase in H_2_O_2_ content (Fig. 2e–h). Therefore, we wanted to determine the effect of exogenous H_2_O_2_ on root growth in *Arabidopsis*. As shown in Fig. 3a, an increase in H_2_O_2_ concentration inhibited the root growth of *Arabidopsis* seedlings. Moreover, this inhibitory effect was positively correlated with the H_2_O_2_ concentration. The 7 DAG and 21 DAG seedlings showed the same trend. The H_2_O_2_ scavenger DMTU partly recovered root growth at lower H_2_O_2_ concentrations (Fig. 3b).

### Effects of exogenous H_2_O_2_ on the dynamics and distribution of actin filaments in Arabidopsis roots

**Fig. 4:**
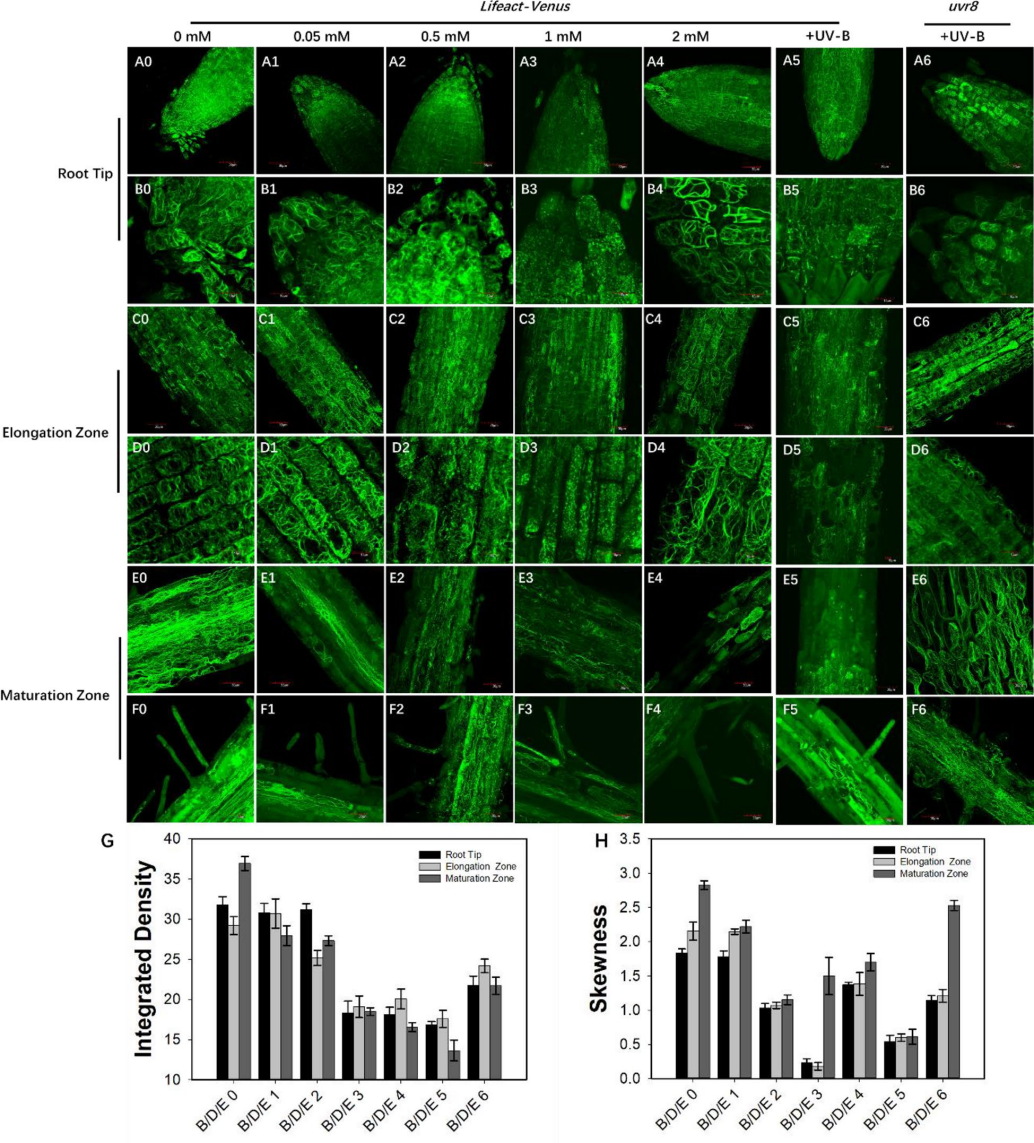
Exogenous H_2_O_2_ changed the arrangement of actin filaments in *Arabidopsis* roots. A0–F0: Control group. A1–F1: 0.05 mM H_2_O_2_ treatment group. A2–F2: 0.5 mM H_2_O_2-_treatment group. A3–F3: 1 mM H_2_O_2_ treatment group. A4–F4: 2 mM H_2_O_2_ treatment group. A0–A6: The distribution of actin filaments in root tips treated with different concentrations of H_2_O_2_ and UV-B in the Lifeact-Venus and *uvr8* lines. B0–B6: A partial enlargement of panels A0–A6. C0–C6: The change in actin filaments in the elongation zone after treatment with different concentrations of H_2_O_2_ and UV-B in the Lifeact-Venus and *uvr8* lines. D0–D6: Partial enlargements of panels C0–C6. E0–E6: Effects of exogenous H_2_O_2_ treatment and UV-B on actin filaments in the mature region of Arabidopsis roots. F0–F6: A partial enlargement of panels E0–E6. Scale bar = 30 μm (A0–A6, C0–C6, E0–E6); 10 μm (B0–B6, D0–D6, F0–F6). **g**, **h** Statistics on the integrated density and skewness in each group

As shown in Fig. 4, panels A0-F6, 7 DAG *Arabidopsis* seedlings (Lifeact-Venus and *uvr8*) were treated with different concentrations of exogenous H_2_O_2_ and UV-B, and the distribution of actin filaments in the roots was observed by confocal microscopy. The morphology of the root tip in response to different H_2_O_2_ treatments was similar to that observed in the absence of UV-B exposure (Fig. 4A0–A4), and the fluorescence signal at the root tip was stronger than that in other regions of the root. The green signal was significantly weakened by treatment with 1 mM exogenous H_2_O_2_ (Fig. 4A3). The morphology of the actin filaments in different groups was much clearer after enlargement, as shown in Fig. 4B0–B4. In the control and 0.05 mM exogenous H_2_O_2_ treatment groups, the apical actin filaments were obviously filamentous (Fig. 4B0, B1). Under the 0.5 mM exogenous H_2_O_2_ treatment, the actin filaments disaggregated into bright puncta (Fig. 4B2). In the 1 mM H_2_O_2_ treatment, almost all the actin filaments disaggregated into puncta (Fig. 4B3). After treatment with the highest concentration of 2 mM exogenous H_2_O_2_, the actin filaments in the root tip changed into a thick bundle-like structure (Fig. 4B4). This suggested that the cell ductility is greatly restricted under treatment with high concentrations of H_2_O_2_. In Fig. 4B0–B4, the actin filaments first depolymerized and then aggregated into a thicker bundle-like structure with increasing H_2_O_2_ concentrations. This may be related to the resistance of plants to stresses induced by external H_2_O_2_. After UV-B treatment, actin filaments in the root tips underwent depolymerization (Fig. 4A5, B5), similar to the results obtained after exogenous 1 mM H_2_O_2_ treatment in the Lifeact-Venus line. In contrast, actin filaments in root apical cells remained filamentous under UV-B exposure in the *uvr8* line (Fig. 4A6, B6). This result indicates that the UV-B-induced change in actin filament morphology in root tip cells is dependent on H_2_O_2_ generation.

The distribution of actin filaments in the elongation zone under different treatments was also observed and analyzed (Fig. C0–D6). Only treatment with 1 mM exogenous H_2_O_2_ showed lower fluorescence intensity in the elongation zone under low magnification (Fig. 4C3). The enlarged results showed that the arrangement of actin filaments in the elongation zone of the control group was diffuse, and the arrangement direction was transverse (Fig. 4C0, D0). After treatment with 0.05 mM H_2_O_2_, the arrangement of the actin filaments was similar to that of the control group (Fig. 4C1, D1). When the exogenous H_2_O_2_ concentration was increased to 0.5 mM, the intracellular actin filaments changed from filaments to puncta (Fig. 4C2, D2). When the concentration reached 1 mM, the number of puncta was the highest (Fig. 4C3, D3), and the presence of filamentous actin was not observed. Under 2 mM H_2_O_2_ treatment, the actin filaments were a thick bundle (Fig. 4C4, D4), and the direction was parallel to the longitudinal axis of the cells (Fig. 4D4). The changes in the distribution of actin filaments in the elongation zone were similar to the changes in the apical area. After UV-B treatment, the actin filaments in cells in the root elongation zone showed both short filamentous structures and more punctate structures in the Lifeact-Venus line (Fig. 4C5, D5). In the *uvr8* mutant, the distribution of microfilaments in the cells in the elongation zone (Fig. 4C6, D6) was similar to that of the control without any treatment (Fig. 4C0, D0).

Finally, we observed and analyzed the response of exogenous H_2_O_2_ to actin filaments in the mature region of the root. The actin filaments in the mature region of the control group (Fig. 4E0) were filamentous, and the 0.05 mM exogenous H_2_O_2_ treatment group (Fig. 4E1) was not significantly different from the control. In the 0.5 mM H_2_O_2_ treatment group (Fig. 4E2, F2), a large number of actin filaments were distributed as green puncta. While the fluorescence intensity of the actin filaments in the 1 mM H_2_O_2-_treated group was much weaker, only partially punctate actin was visible (Fig. 4E3). This result also implies that the mature region is more sensitive to ROS accumulation. After treatment with a high concentration of H_2_O_2_, green fluorescence could be detected in several cells in the short mature region, and the actin filaments gathered in a thick bundle (Fig. 4E3, F4). UV-B treatment resulted in complete degradation of actin filaments in the cells in the root maturation zone, with no filamentous or punctate structures present in the Lifeact-Venus line (Fig. 4E5, F5). In contrast, cells in the root maturation zone of the *uvr8* mutant still showed more microfilament bundles (Fig. 4E6, F6).

We analyzed the integrated density after different treatments and in different root regions, as shown in Fig. 4g. The results showed that the integrated density in the Lifeact-Venus line continued to decrease with increasing exogenous H_2_O_2_ concentrations. The integrated density also greatly diminished after exposure to UV-B in the Lifeact-venus line. In contrast, the overall integrated density of the *uvr8* mutant roots under UV-B exposure remained high. The skewness results (Fig. 4h) showed that 1 mM exogenous H_2_O_2_ increased skewness in the maturation zone, which showed the same tendency as *uvr8*. This means that the maturation zone could be a region of signal transduction in the regulation of actin filament dynamics.

## Discussion

In this study, we first screened transgenic lines for the distribution and dynamics of actin filaments. We found that transformed *fABD2-GFP* is mainly expressed in the aboveground parts of seedlings, especially in leaf epidermal cells and stomatal guard cells (Fig. 1a–e). In the transformed Lifeact-Venus line, it was mainly expressed in the underground part of the seedling, especially in the root of the whole seedling (Fig. 1k–o). To reflect the distribution of actin filaments in the roots more accurately, the Lifeact-Venus line was chosen for subsequent research. Researchers have compared these two lines to show the distribution of actin filaments. They found that the *fABD2-GFP* line showed thicker bundles that appeared as a highly dynamic remodeling meshwork. The Lifeact-Venus line showed much sparser thick bundles [33]. The reason for the difference in this paper could be the timing of material selection. However, we found a difference in the expression of the two lines at the seedling stage after comparison.

Studies on UV-B-mediated inhibition of hypocotyl elongation in *Arabidopsis* are more common, especially studies focused on the UV-B receptor. However, there is no report on whether this inhibitory effect is related to H_2_O_2_ production in the hypocotyl. Here, we found no significant H_2_O_2_ accumulation in the hypocotyl before or after UV-B treatment in either Lifeact-Venus or *uvr8* (Fig. 2e–h). However, ROS changes were obvious in the root region, especially the H_2_O_2_ content, which was greatly improved. A previous study showed that UV-B causes an increase in plant ROS [9]. However, changes in the ROS burst in hypocotyls that were inhibited have not been reported thus far. This is a very interesting phenomenon, suggesting that the inhibition of hypocotyl elongation may not be related to the important signaling molecule H_2_O_2_. UVR8 is important in hypocotyl elongation. It is possible that the UVR8 signaling pathway affects the downstream molecules HY5 and so on and ultimately affects the expression of growth-related molecules. However, the specific reasons for this are worthy of further in-depth research, and it is possible that a new pathway that regulates growth and is independent of ROS will be uncovered.

Moreover, Dyachok reported that light-activated COP1, an E3 ubiquitin ligase, promotes actin polymerization and actin filament bundling through regulation of the downstream ARP2/3-SCAR pathway in root cells [34]. COP1 promoted actin filament aggregation under normal light conditions (Fig. 2i). The UVR8 signaling pathway is activated by treatment with UV-B, and monomer UVR8 combines with COP1 in the nucleus. Therefore, COP1 no longer promotes the aggregation of actin filaments, in which case actin filaments disaggregate (Fig. 2k). However, we found that *uvr8* also showed a high level of skewness (Fig. 2p), which suggested that there could be another factor invovled in regulating the dynamics of actin filaments in hypocotyls.

Four different concentrations of H_2_O_2_ were added to MS medium as exogenous processing molecules. Seedlings at both 7 DAG and 21 DAG showed the same suppression of root growth (Fig. 3). The inhibitory effect was positively correlated with the concentration of H_2_O_2_ added. However, DMTU partially recovered the inhibition of root growth under exogenous H_2_O_2_ treatment. Observation of the arrangement of actin filaments also revealed an interesting phenomenon (Fig. 4). The actin filaments tended to disaggregate into puncta in response to exogenous H_2_O_2_ concentrations less than 1 mM and UV-B exposure, while they tended to aggregate into a thicker bundle-like structure at 2 mM. Therefore, the behavior of actin filaments under different concentrations of H_2_O_2_ can be summarized as disintegration of fine filaments into puncta and subsequent aggregation of actin filaments in thick bundles. The change in the actin filament response to exogenous H_2_O_2_ could be related to the adaptation of plants to external stress stimuli to regulate cell metabolic activities and improve plant survival. The actin filaments tended to depolymerize under UV-B exposure, which was similar to the results obtained after exogenous H_2_O_2_ treatment. The *uvr8* mutant, on the other hand, showed the opposite pattern. This implies that the dynamics of microfilaments in root cells under UV-B exposure are strongly related to H_2_O_2_ production.

It has been reported that the rearrangement of actin filaments is an important factor for root elongation [20]. An actin-dependent mechanism that controls the relative vacuolar occupancy of the cell for cytosol homeostasis during cellular growth has been confirmed [31]. Studies have pointed out that exogenous H_2_O_2_ can disrupt the normal distribution of actin filaments in plants. Our finding of the change in actin filaments from disaggregation to reassembly may inspire people to understand the importance of actin filaments in terms of cell function and plant adaptation.

**Fig. 5:**
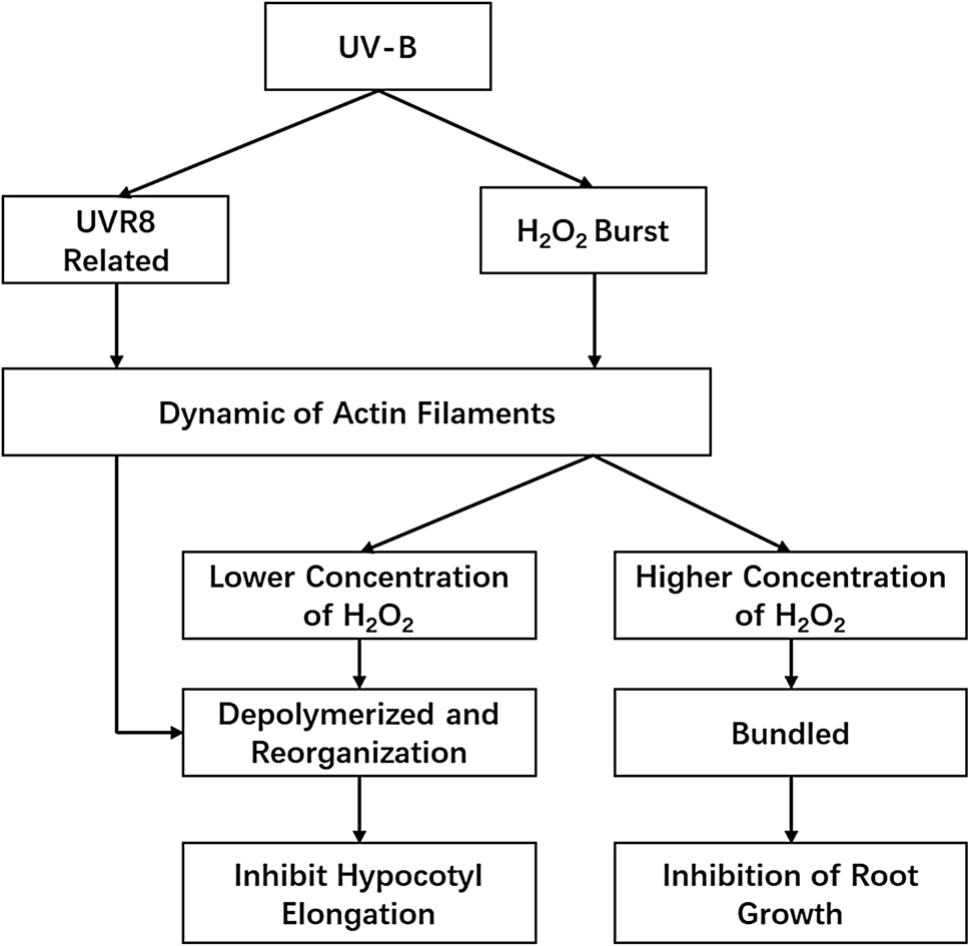
The mechanisms by which actin filaments are involved in plant growth inhibition under UV-B exposure

A possible mechanism underlying the inhibitory response of hypocotyls and roots to UV-B exposure is shown in Fig. 5. The response of Arabidopsis to UV-B may occur in two ways. One way involves inhibition of the elongation of hypocotyls via a UVR8-dependent pathway. COP1 and other downstream molecules may affect the dynamic changes of actin filaments and change the distribution of actin filaments in the hypocotyl. The other way involves the induction of ROS accumulation and changes in the arrangement of actin filaments in different regions, which inhibits root growth. The dynamic change in actin filaments is related to the concentration of H_2_O_2_. At lower concentrations, the actin filaments tend to disaggregate into puncta. At higher concentrations, the actin filaments tend to aggregate into thicker microfilaments. These changes may be a strategy by which plants to adapt to the environment.

## Conclusion

Plant roots show growth inhibition under different environmental stimuli. Under UV-B exposure, the hypocotyl elongation and root growth of *Arabidopsis* seedlings were inhibited. Our research showed that actin filaments depolymerized and reassembled in hypocotyls without H_2_O_2_ production when exposed to UV-B. The inhibition of root growth was mainly due to the reorganization of actin filaments by the accumulation of H_2_O_2_ under UV-B exposure. Therefore, UV-B affected the dynamics of actin filaments in different ways during hypocotyl elongation and root growth. These results provide an experimental basis for further understanding the important functions of actin filaments, which could be downstream functional molecules in UV-B signaling.

## Data Availability

The data could be obtained upon request to the corresponding author.

## Abbreviations

UV-B: Ultraviolet B
UVR8: UV resistance locus 8
COP1: Constitutively photomorphogenic 1
HY5/HYH: Elongated hypocotyl 5/hy5 related homolog
WRKY36: WRKY DNA-binding protein 36
BIM1: BES1-interacting MYC-like 1
BES1: BRI1-EMS-suppressor 1
MYB73/MYB77: MYB domain protein 73/77
ROS: Reactive oxidative species
NO: Nitric oxide
DMTU: Dimethylthiourea

## References

[1] Jenkins GI (2009). Signal transduction in responses to UV-B radiation. Annu Rev Plant Biol.

[2] Noble RE (2002). Effects of UV-irradiation on seed germination. Sci Total Environ.

[3] Rizzini L, Favory J-J, Cloix C, Faggionato D, O’Hara A, Kaiserli E, Baumeister R, Schäfer E, Nagy F, Jenkins GI (2011). Perception of UV-B by the *Arabidopsis* UVR8 protein. Science.

[4] Liang T, Yang Y, Liu H (2018). Signal transduction mediated by the plant UV-B photoreceptor UVR8. New Phytol.

[5] Liang T, Mei S, Shi C, Yang Y, Peng Y, Ma L, Wang F, Li X, Huang X, Yin Y, Liu H (2018). UVR8 interacts with BES1 and BIM1 to regulate transcription and photomorphogenesis in *Arabidopsis*. Dev Cell.

[6] Liang T, Shi C, Peng Y, Tan H, Xin P, Yang Y, Wang F, Li X, Chu J, Huang J, Yin Y, Liu H. Brassinosteroid-Activated BRI1-EMS-SUPPRESSOR 1 inhibits flavonoid biosynthesis and coordinates growth and UV-B stress responses in plants*.* Plant Cell. 2020: tpc.00048.2020. 10.1105/tpc.20.00048 PMC7534464 32796123

[7] Yang Y, Zhang L, Chen P, Liang T, Li X, Liu H (2019). UV-B photoreceptor UVR8 interacts with MYB73/MYB77 to regulate auxin responses and lateral root development. EMBO J.

[8] Vanhaelewyn L, Viczian A, Prinsen E, Bernula P, Serrano AM, Arana MV, Ballare CL, Nagy F, Van Der Straeten D, Vandenbussche F (2019). Differential UVR8 signal across the stem controls UV-B induced inflorescence phototropism. Plant Cell.

[9] Yokawa K, Kagenishi T, Baluska F (2015). UV-B induced generation of reactive oxygen species promotes formation of BFA-induced compartments in cells of *Arabidopsis* root apices. Front Plant Sci.

[10] Schuh M (2011). An actin-dependent mechanism for long-range vesicle transport. Nat Cell Biol.

[11] Pollard TD, Cooper JA (2009). Actin, a central player in cell shape and movement. Science.

[12] Plessner M, Grosse R (2018). Dynamizing nuclear actin filaments. Curr Opin Cell Biol.

[13] Iwabuchi K, Ohnishi H, Tamura K, Fukao Y, Furuya T, Hattori K, Tsukaya H, Hara-Nishimura I (2019). ANGUSTIFOLIA regulates actin filament alignment for nuclear positioning in leaves. Plant Physiol.

[14] Svitkina TM (2018). Ultrastructure of the actin cytoskeleton. Curr Opin Cell Biol.

[15] Wada M, Kong SG (2018). Actin-mediated movement of chloroplasts. J Cell Sci.

[16] Qu X, Zhang R, Zhang M, Diao M, Xue Y, Huang S (2017). Organizational innovation of apical actin filaments drives rapid pollen tube growth and turning. Mol Plant.

[17] Ketelaar T (2013). The actin cytoskeleton in root hairs: all is fine at the tip. Curr Opin Plant Biol.

[18] Chen H, Han R (2015). F-actin participates in the process of the “partition-bundle division”. Russ J Plant Physiol.

[19] Zou M, Ren H, Li J (2019). An auxin transport inhibitor targets Villin-mediated actin dynamics to regulate polar auxin transport. Plant Physiol.

[20] Takatsuka H, Higaki T, Umeda M (2018). Actin reorganization triggers rapid cell elongation in roots. Plant Physiol.

[21] Qian D, Xiang Y (2019). Actin cytoskeleton as actor in upstream and downstream of calcium signaling in plant cells. Int J Mol Sci.

[22] Plohovska SH, Krasylenko YA, Yemets AI (2019). Nitric oxide modulates actin filament organization in *Arabidopsis thaliana* primary root cells at low temperatures. Cell Biol Int.

[23] Waszczak C, Carmody M, Kangasjarvi J (2018). Reactive oxygen species in plant signaling. Annu Rev Plant Biol.

[24] Liu SG, Zhu DZ, Chen GH, Gao X-Q, Zhang XS (2012). Disrupted actin dynamics trigger an increment in the reactive oxygen species levels in the *Arabidopsis* root under salt stress. Plant Cell Rep.

[25] Nick P (2010). Stress, ROS, and actin—a volatile menage a trois?. Protoplasma.

[26] Chen H, Han R (2016). Characterization of actin filament dynamics during mitosis in wheat protoplasts under UV-B radiation. Sci Rep.

[27] Lee H-J, Ha J-H, Kim S-G, Choi H-K, Kim ZH, Han Y-J, Kim J-I, Oh Y, Fragoso V, Shin K (2016). Stem-piped light activates phytochrome B to trigger light responses in Arabidopsis thaliana roots. Sci Signal.

[28] Voigt B, Timmers AC, Samaj J, Muller J, Baluska F, Menzel D (2005). GFP-FABD2 fusion construct allows in vivo visualization of the dynamic actin cytoskeleton in all cells of *Arabidopsis* seedlings. Eur J Cell Biol.

[29] Era A, Tominaga M, Ebine K, Awai C, Saito C, Ishizaki K, Yamato KT, Kohchi T, Nakano A, Ueda T (2009). Application of Lifeact reveals F-actin dynamics in *Arabidopsis thaliana* and the liverwort. Marchantia Polym Plant Cell Physiol.

[30] De Zacchini M, De Agazio M (2001). Dimethylthiourea, a hydrogen peroxide trap, partially prevents stress effects and ascorbate peroxidase increase in spermidine-treated maize roots. Plant Cell Environ.

[31] Scheuring D, Lofke C, Kruger F, Kittelmann M, Eisa A, Hughes L, Smith RS, Hawes C, Schumacher K, Kleine-Vehn J (2016). Actin-dependent vacuolar occupancy of the cell determines auxin-induced growth repression. Proc Natl Acad Sci USA.

[32] Higaki T, Kutsuna N, Sano T, Kondo N, Hasezawa S (2010). Quantification and cluster analysis of actin cytoskeletal structures in plant cells: role of actin bundling in stomatal movement during diurnal cycles in *Arabidopsis* guard cells. Plant J.

[33] Tolmie F, Poulet A, McKenna J, Sassmann S, Graumann K, Deeks M, Runions J (2017). The cell wall of *Arabidopsis thaliana* influences actin network dynamics. J Exp Bot.

[34] Dyachok J, Zhu L, Liao F, He J, Huq E, Blancaflor EB (2011). SCAR mediates light-induced root elongation in Arabidopsis through photoreceptors and proteasomes. Plant Cell.

